# Abnormal functional connectivity of the core olfactory network in patients with chronic rhinosinusitis accompanied by olfactory dysfunction

**DOI:** 10.3389/fneur.2023.1295556

**Published:** 2023-11-16

**Authors:** Yao Ma, Jian Jiang, Ying Wu, Jiaxin Xiong, Huiting Lv, Jiahao Li, Hongmei Kuang, Xiaofeng Jiang, Yeyuan Chen

**Affiliations:** ^1^Department of Radiology, The First Affiliated Hospital, Nanchang University, Nanchang, China; ^2^Department of Radiology, The Third Hospital of Nanchang, Nanchang, Jiangxi, China; ^3^Department of Radiology, The Second Affiliated Hospital, Nanchang University, Nanchang, China; ^4^Department of Neurology, The First Affiliated Hospital of Xi'an Jiaotong University, Xi'an, Shaanxi, China

**Keywords:** functional connectivity, olfactory network, chronic rhinosinusitis, olfactory dysfunction, functional magnetic resonance imaging

## Abstract

**Objective:**

To review and analyze the functional connectivity (FC) abnormalities in the brain olfactory network (ON) of patients with chronic rhinosinusitis with olfactory dysfunction (CRSwOD) and explore the relationship between these FC abnormalities and olfactory dysfunction, providing clues to the neurophysiological mechanisms underlying CRSwOD.

**Methods:**

FC analysis on the ON of patients with CRSwOD and patients with chronic rhinosinusitis without olfactory dysfunction (CRSsOD) identified the regions of the ON with abnormal FC in CRSwOD patients, and the correlation between abnormal FC and clinical scales for chronic rhinosinusitis was analyzed.

**Results:**

(1) Compared with the CRSsOD group, CRSwOD patients showed decreased FC between the bilateral orbitofrontal cortex (OFC) and the right middle frontal gyrus, (2) Receiver operating characteristic (ROC) curve analysis revealed that the FC value between the right middle frontal gyrus and the left OFC (area under the curve (AUC) = 0.852, sensitivity: 0.821, specificity: 0.800, *p* < 0.001) was more capable of distinguishing whether CRS patients may have olfactory dysfunction than the FC value between the right middle frontal gyrus and the right OFC (AUC = 0.827, sensitivity: 0.893, specificity: 0.667, *p* < 0.001), and (3) Lund-Kennedy scores were positively correlated with the FC values between the right middle frontal gyrus and the left OFC (*r* = 0.443, *p* < 0.018). Lund-Mackay scores were also positively correlated with the FC values between the right middle frontal gyrus and the left OFC (*r* = 0.468, *p* < 0.012). Questionnaire of Olfactory Disorders-Negative Statements scores were negatively correlated with the FC values between the right middle frontal gyrus and the left OFC (*r* = −0.481, *p* < 0.001).

**Conclusion:**

Persistent nasal inflammation affects the FC between the middle frontal gyrus and the OFC, which may serve as a potential imaging marker for identifying CRSwOD. The severity of nasal inflammation and olfactory damage is closely related to the FC between the middle frontal gyrus and OFC, and the abnormal changes in this FC can be used to explain the neurophysiological mechanisms behind the occurrence of olfactory dysfunction in patients.

## 1 Introduction

Chronic rhinosinusitis (CRS) is an inflammatory disease characterized by persistent nasal congestion, nasal discharge, facial pain, and olfactory dysfunction (OD). The overall prevalence of OD in the CRS population is approximately 67% (1). OD has a significant negative impact on the quality of life of patients. First, it may affect appetite and eating behavior, as smell plays a crucial role in the taste and enjoyment of food (2). Second, individuals may face safety issues due to their inability to detect warning odors such as smoke or gas leaks. Third, OD can affect personal social activities and emotional health, leading to anxiety, depression, and difficulty in social interactions (3). Overall, OD can decrease patient satisfaction and overall quality of life (4).

Olfactory information is detected by olfactory neurons in the olfactory epithelium, and the axons directly transmit information to the olfactory bulb in the brain, which subsequently projects to other cortical areas, including the primary and secondary olfactory cortex (5). Specifically, the olfactory cortex is composed of the piriform cortex, the anterior and lateral parts of the amygdala cortex, and the ventral medial prefrontal cortex. The secondary olfactory cortex includes the orbitofrontal cortex (OFC), insula, anterior cingulate cortex, striatum, thalamus, superior temporal gyrus, and hippocampus, which collectively make up the brain’s olfactory network (6). The core olfactory network includes the OFC and the piriform area of the anterior temporal cortex, extending to the amygdala and the contextual memory system (medial temporal lobe, frontal lobe, and hippocampus) (7).

Magnetic resonance imaging (MRI) provides a powerful tool for studying the neurostructural and functional changes in patients with OD. Relevant studies have shown that inflammation plays a dominant role in CRS with OD (CRSwOD), causing OD by disrupting the mucosal microenvironment and olfactory sensory neurons in the olfactory region (8). Prolonged inflammation and mucus accumulation hinder the arrival of odor molecules to the olfactory neurons, resulting in a decline in olfactory ability. If olfactory input is continuously absent or weakened, changes may occur in the olfactory network of the brain (4). For example, in coronavirus disease 2019 (COVID-19) cases with long-term olfactory deficiency, mild reductions in gray matter thickness (ranging from 0.2 to 2%) were found in the OFC and the adjacent hippocampal gyrus, and there was an increase in tissue damage markers in the functional connectivity (FC) between the piriform cortex and related areas (9), which may be due to neural plasticity changes caused by a prolonged lack of olfactory stimulation. Another study found that there was a significant increase in gray matter volume in the secondary olfactory network, including the hippocampus and the parahippocampal gyrus on the right side, in patients with improved olfactory dysfunction symptoms after surgical treatment of CRS (10), further confirming the plasticity changes in the central olfactory network structure.

However, the functional changes in the brain in CRSwOD patients are still unclear. Further exploration of the alterations in the olfactory network in CRSwOD patients is needed. It has been observed that the severity of olfactory impairment reflects the olfactory network, which affects its FC (11). In patients with persistent OD related to COVID-19, the overall modular coefficient of the olfactory network was significantly decreased compared to control subjects, and the number and strength of functional connections centered on the right thalamus were significantly increased (12). In patients with Parkinson’s disease with OD, there are abnormal connections between specific gray matter regions (brainstem, right cerebellum, and right superior temporal lobe) and white matter fibers (left thalamic radiation and bilateral posterior coronal radiation), and there is a strong correlation between the brainstem and right cerebellum (13). In patients with Alzheimer’s disease with olfactory identification dysfunction, the FC between the right OFC and the right frontal/central gyrus was greater than that in the control group, and the bilateral piriform area FC was abnormal in patients with severe olfactory identification dysfunction (14). However, there is currently a lack of reliable evidence of changes in the FC of the brain olfactory network in CRSwOD patients.

Therefore, this study aimed to calculate the FC between the core olfactory network and other regions in CRS without OD (CRSsOD) patients and CRSwOD patients and to identify abnormal functional connections in the olfactory network of CRSwOD patients. The overall objectives of this study were (i) to determine the abnormal functional connections related to the olfactory network in CRSwOD patients; (ii) to identify the functional connections that can diagnose CRSwOD effectively; and (iii) to explore the relationship between the abnormal functional connections in the olfactory network of CRSwOD patients and the clinical indicators related to olfaction.

## 2 Materials and methods

A total of 28 CRSwOD and 29 CRSsOD subjects were recruited from the Department of Otolaryngology-Head and Neck Surgery, the First Affiliated Hospital of Nanchang University. The inclusion criteria were as follows: (1) age 18–55 years old; (2) diagnosed with bilateral CRS with or without olfactory impairment according to the 2020 European Position Paper on Rhinosinusitis and Nasal Polyps; (3) right-handed; and (4) capable of cooperating with olfactory assessment and imaging examinations. The exclusion criteria were as follows: (1) history of olfactory and gustatory dysfunction; (2) history of head trauma, allergic rhinitis and surgery for CRS; (3) history of neurological disorders or other serious illnesses; (4) psychiatric disorders; (5) contraindications for MRI scanning; and (6) visible brain imaging abnormalities. Before MRI image acquisition, patients were screened for severe depression using the Patient Health Questionnaire-2 (PHQ-2). In addition, all subjects underwent standard otolaryngoscopic examination, sinus computed tomography (CT), and olfactory testing and completed the Questionnaire of Olfactory Disorders-Negative Statements (QOD-NS). Written informed consent was obtained from all subjects before data collection. This study was conducted in accordance with the approved guidelines and principles of the Helsinki Declaration. The study was also approved by the Medical Research Ethics Committee and Institutional Review Board of the First Affiliated Hospital of Nanchang University.

### 2.1 Clinical indicators

The QOD-NS consists of 17 negative statements about the extent of olfactory impairment in patients, with lower scores indicating a poorer olfactory life experience (15). The PHQ-2 primarily assesses the patient’s depressive state (with a total score ranging from 0 to 6), with a score of ≥3 indicating severe depression (16). The Lund-Kennedy endoscopic score (LKES) uses the Lund-Kennedy (LK) scoring criteria, with higher scores indicating more severe nasal mucosal inflammation (17). The Lund-Mackay (LM) score for the nasal sinus CT grading system divides the nasal sinuses into the maxillary sinuses; anterior ethmoid, posterior ethmoid, and sphenoid sinuses; frontal sinuses; and the osteomeatal complex and then assigns scores based on their CT appearance, with higher scores indicating a higher grade of polyps and a wider surgical scope (18).

### 2.2 Olfactory testing

Objective assessment of olfactory function was performed using the Sniffin’ Sticks test (Burghart Messtechnik, Wedel, Germany) to evaluate the bilateral olfactory detection threshold (T), odor discrimination (D), and odor identification (I), with each threshold scale ranging from 1 to 16. The TDI score is the sum of the three components, and olfactory function is classified as follows: normal olfaction, ≥30.5; hyposmia, between 16.5 and 30.5; and anosmia, <16.5 (19).

### 2.3 MRI data acquisition

MRI data were acquired using a 3.0 T MRI system (Discovery MR750; GE Healthcare, Milwaukee, WI). Resting-state functional MRI (fMRI) data were obtained using an echo-planar imaging sequence with the following parameters: repetition time (TR) = 2000 ms; echo time (TE) = 30 ms; flip angle = 90°; matrix = 64 × 64; field of view (FOV) = 220 × 220 mm; slice thickness = 4 mm; 240 time points. The high-resolution anatomical 3D T1 imaging sequence parameters were as follows: TR = 1900 ms; TE = 2.26 ms; flip angle = 9°; matrix = 240 × 256; FOV = 215 × 230 mm; slice thickness = 1.0 mm; 176 sagittal slices. Conventional T2-weighted imaging was performed to rule out visible brain structural abnormalities. During the scanning process, all subjects were instructed to keep their heads still, remain awake, and wear earplugs to reduce noise from the MRI machine.

### 2.4 Resting-state fMRI data preprocessing

Resting-state fMRI data were preprocessed using the Resting-State fMRI Data Analysis Toolkit (DPARSF_v5.3, http://rfmri.org/DPARSF) and Statistical Parametric Mapping software (SPM12, Wellcome Department of Imaging Neuroscience, London, United Kingdom) in MATLAB R2018b (The MathWorks Inc., Natick, MA) (20). The specific steps included (1) removing the first 10 time points of each image to ensure the stability of the magnetic field during MRI; (2) slice timing correction using the middle slice as a reference to eliminate the temporal differences caused by interslice acquisition and ensure the same starting point for the collected images; and (3) coregistration of functional and anatomical images, spatial normalization to the Montreal Neurological Institute (MNI) template, and resampling of each voxel to a voxel size.

### 2.5 Definition of nodes

**Figure 1:**
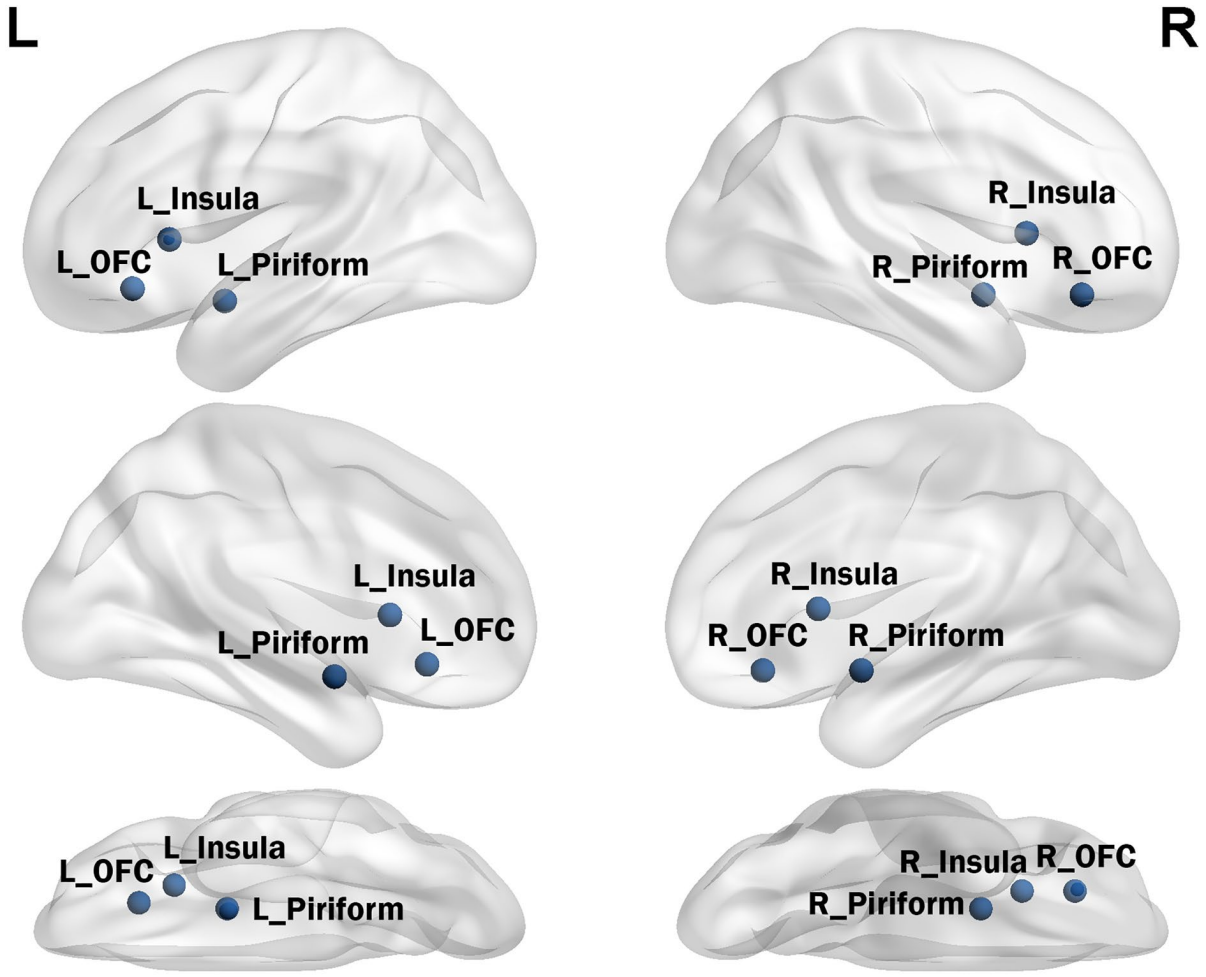
Core seed points of the olfactory network.

According to previously published fMRI task activation studies, the olfactory network includes the olfactory cortex, insula, and OFC (21, 22). Seed time series were extracted from preprocessed data in MNI space (x, y, and z coordinates) as the average time series within a five-voxel radius around the coordinates defined in previous activation studies (see Figure 1). The seed for the core olfactory network was derived from meta-analyses that identified three bilateral brain regions as most likely to be activated by olfactory stimuli, including the olfactory cortex ([−22 0–14], [22 2–12]), orbitofrontal cortex ([−24 30–10], [28 34–12]), and insula ([−30 18 6], [28 16 8]). The network was computed as the FC map surviving at *p* < 0.01 and a minimum cluster size (*k*) of 60 voxels.

### 2.6 Static functional connectivity

The static FC was quantified using the Pearson correlation coefficient (*r*) between the average time series of each region of interest (ROI) voxel and the average time series of all other voxels in the brain. Subsequently, Fisher’s Z transformation was used to convert all *r* values to *z* values.

### 2.7 Statistical analysis

Population demographics were analyzed using SPSS 17.0. The Shapiro–Wilk test was used to assess the normality of continuous variables, and normally distributed continuous variables are presented as the mean ± standard deviation, while nonnormally distributed continuous variables are presented as the median ± interquartile range. Group differences were assessed using two-sample *t* tests, and sex proportions were compared using chi-square tests. To explore the differences in FC between patients with CRSwOD and patients with CRSsOD, independent two-sample *t* tests were performed on Fisher’s Z transformed scores, using Gaussian random field (GRF) correction.

Then we generated the receiver operating characteristic (ROC) curve to analyze whether the brain regions with significant differences in FC values between CRSwOD and CRSsOD patients could distinguish these patient groups, and calculated the area under the ROC curve (AUC) to evaluate the diagnostic efficacy.

## 3 Results

### 3.1 Clinical characteristics of participants

A total of 57 participants were recruited, including 29 patients with CRSsOD (17 males, 12 females) and 28 patients with CRSwOD (19 males, 9 females). Other than TDI scores, which were significantly higher in the CRSsOD group than in the CRSwOD group (*p* < 0.05), there were no significant differences in age, sex, or other clinical data, including PHQ-2, QOD-NS, LK, and LM scores, between the two groups (*p* > 0.05). The demographic data of all participants are summarized in Table 1.

**Table 1:** Demographic and clinical data of the CRSsOD and CRSwOD groups.

	CRSsOD (*n* = 29)	CRSwOD (*n* = 28)	*p* value
Age, years	36.33 ± 11.49	42.25 ± 13.99	0.08
Sex, male/female	17/12	19/9	0.19
TDI score	36.23 ± 2.79	13.84 ± 34.67	<0.01
PHQ-2 score	1.69 ± 1.48	1.74 ± 1.45	0.46
QOD-NS score	35.47 ± 10.13	38.43 ± 5.74	0.29
LK score	±2.29	6.00 ± 2.02	0.73
LM score	14.53 ± 5.14	15.39 ± 12.83	0.35

CRSwOD, chronic rhinosinusitis with olfactory dysfunction; CRSsOD, chronic rhinosinusitis without olfactory dysfunction; TDI, the bilateral olfactory detection threshold (T), odor discrimination (D), and odor identification (I); PHQ-2, the Patient Health Questionnaire-2; QOD-NS, the Questionnaire of Olfactory Disorders-Negative Statements; LK score, the Lund-Kennedy endoscopic score; LM score, the Lund-Mackay score.

### 3.2 Differences in resting-state functional connectivity between the CRSsOD and CRSwOD groups

**Figure 2:**
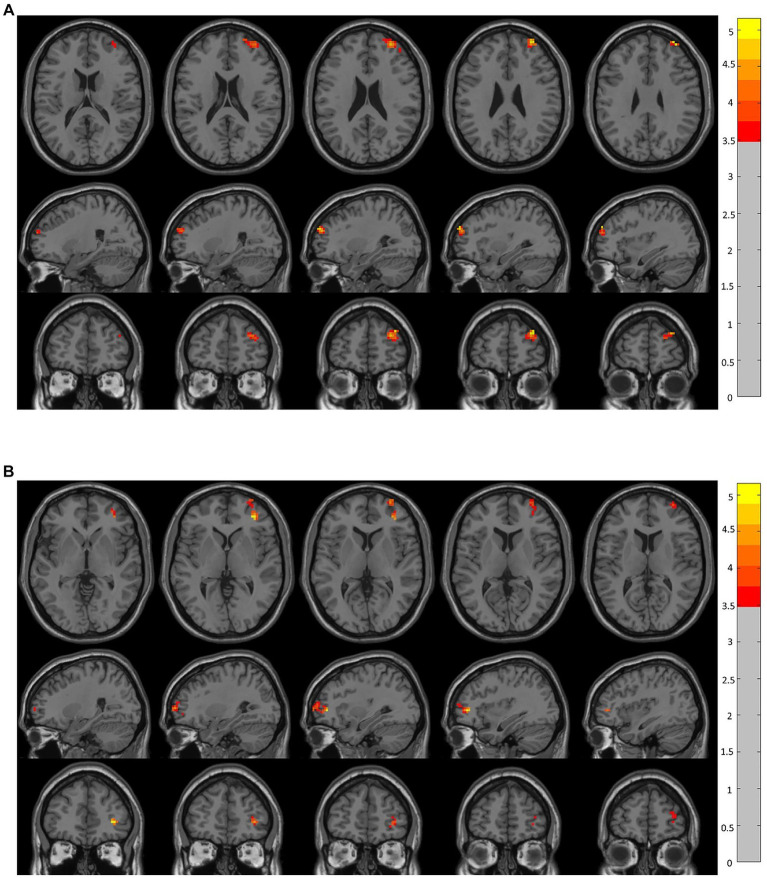
Brain regions showing significant changes in resting-state functional connectivity in the prefrontal cortex (voxel-level *p* < 0.05, GRF corrected). There were significant between-group differences (*p* < 0.05) in the average weighted resting-state functional connectivity in these regions. **(A)** Compared to the CRSwOD group, patients with CRSsOD showed increased functional connectivity between the right orbitofrontal cortex (OFC) and right middle frontal gyrus, with no significant functional connectivity differences observed between other seed points. **(B)** Compared to the CRSwOD group, patients with CRSsOD showed increased functional connectivity between the left OFC and right middle frontal gyrus, with no significant functional connectivity differences observed between other seed points.

**Figure 3:**
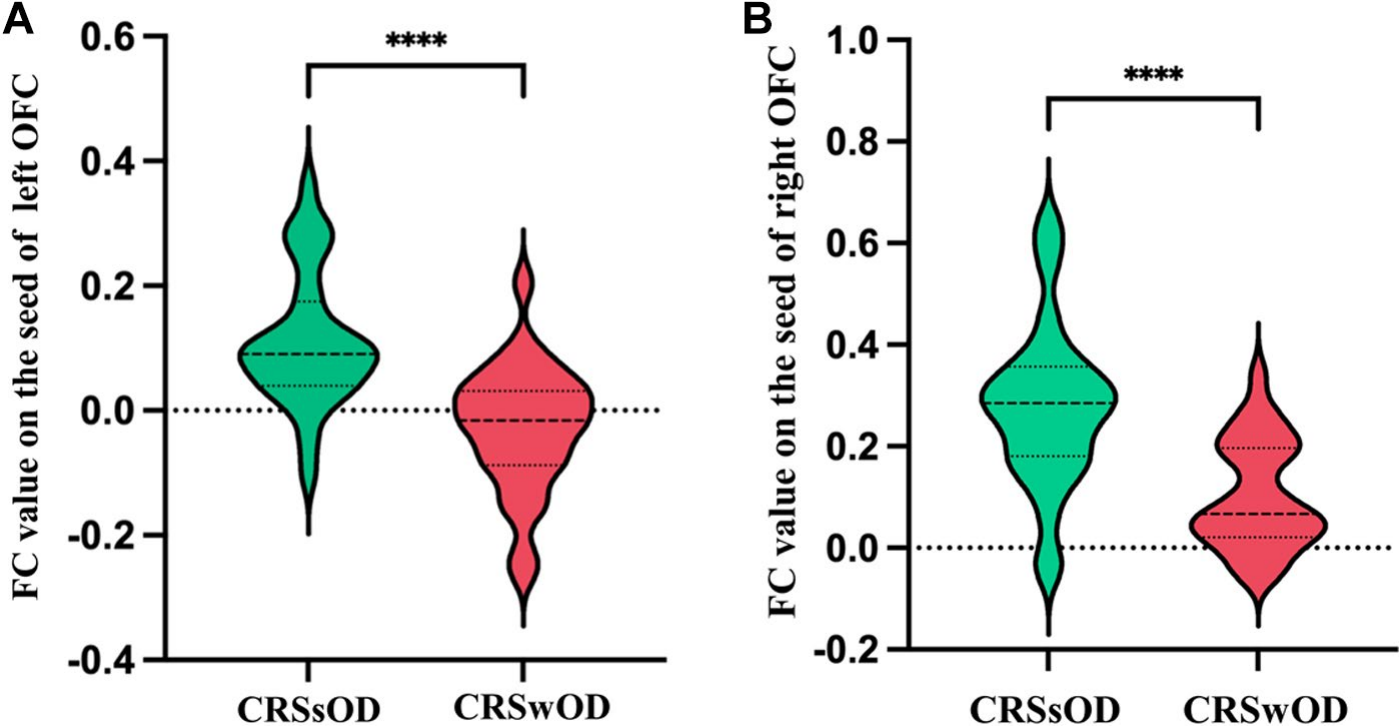
Average abnormal functional connectivity values (*Z* scores) between the left orbitofrontal cortex **(A)** and right orbitofrontal cortex **(B)** in CRSwOD patients compared to CRSsOD patients. * Represents a significant difference, * *p* < 0.05, ** *p* < 0.01, *** *p* < 0.001, **** *p* < 0.0001.

The voxel-level results of intergroup resting-state FC comparisons using the bilateral OFC as the seed region are presented in Table 2. Compared to the CRSwOD group, CRSsOD patients showed significantly increased FC between the bilateral OFC and right middle frontal gyrus (two-sample *t* test, *p* < 0.05, GRF corrected) (Figures 2, 3). However, no significant differences in FC were found between the two groups using the seed regions of piriform cortex and insula lobe.

**Table 2:** Differences in FC values between the CRSsOD and CRSwOD groups.

Seed ROI	L/R	Brain area	Cluster size (voxels)	MNI coordinates of peak voxel			*t* value
				X	Y	Z	
Left OFC							
	R	Frontal-Mid	58	36	54	30	−0.95
Right OFC							
	R	Frontal-Mid	70	36	45	3	−0.76

The coordinates are stereotactic coordinates based on the Montreal Neurological Institute (MNI) brain atlas. OFC, orbitofrontal cortex; Frontal-Mid, frontal middle gyrus.

### 3.3 Receiver operating characteristic curve analysis

**Figure 4:**
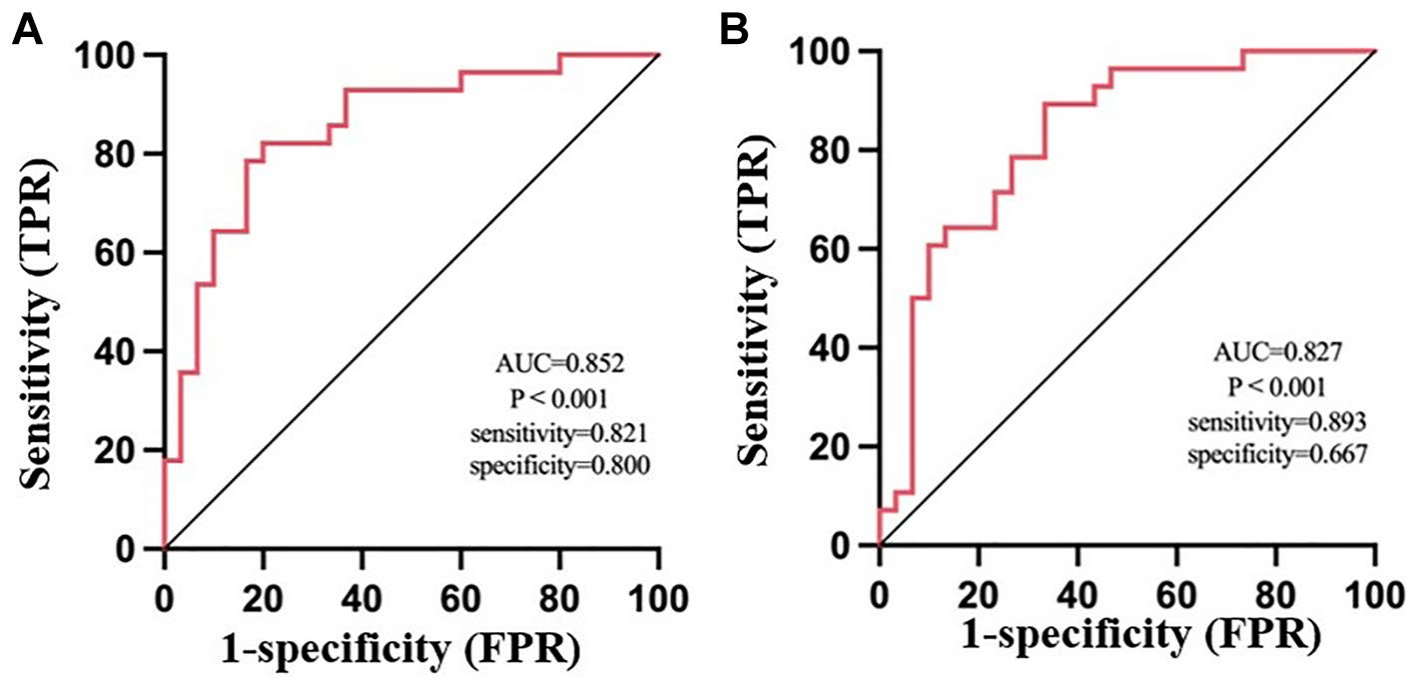
ROC analysis of the average weighted functional connectivity values in the changing brain regions based on the left orbitofrontal cortex **(A)** and right orbitofrontal cortex **(B)** in patients with CRSsOD and CRSwOD. FPR, false-positive rate; TPR, true-positive rate.

The FC values between the right middle frontal gyrus and left OFC (area under the curve (AUC) = 0. 852, sensitivity: 0.821, specificity: 0.800, *p* < 0.001) were more effective in distinguishing olfactory functional changes in CRS patients than the FC values between the right middle frontal gyrus and right OFC (AUC = 0.827, sensitivity: 0.893, specificity: 0.667, *p* < 0.001) (Figure 4).

### 3.4 Correlation analysis

**Figure 5:**
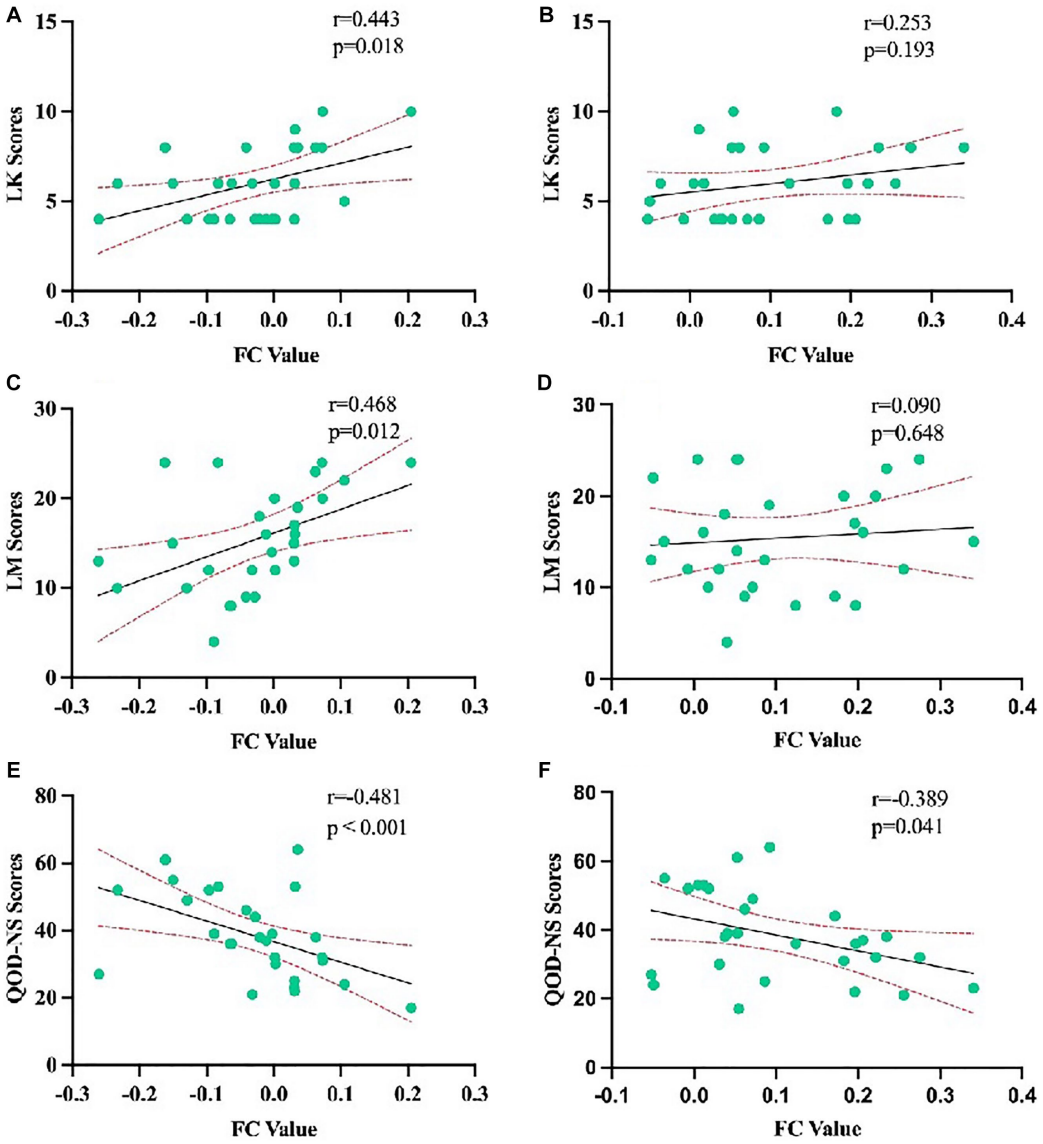
Correlation analysis of LK scores, LM scores, and QOD-NS scores with the average weighted functional connectivity values in the changing brain regions based on the left orbitofrontal cortex in CRSwOD patients **(A,C,E)** and with the average weighted functional connectivity values in the changing brain regions based on the right orbitofrontal cortex in CRSwOD patients **(B,D,F)**.

We analyzed the correlation among LK scores, LM scores, QOD-NS scores, and FC values between the bilateral OFC and right middle frontal gyrus. LK scores were positively correlated with the FC values between the right middle frontal gyrus and left OFC (*r* = 0.443, *p* < 0.018). LM scores were also positively correlated with the FC values between the right middle frontal gyrus and left OFC (*r* = 0.468, *p* < 0.012). QOD-NS scores were negatively correlated with the FC values between the right middle frontal gyrus and left OFC (*r* = −0.481, *p* < 0.001). However, there were no significant correlations among LK scores, LM scores, QOD-NS scores, and FC values between the right middle frontal gyrus and right OFC (Figure 5).

## 4 Discussion

This study investigated the differences in FC between the olfactory network and other brain regions in patients with CRSsOD and CRSwOD. Compared to patients with CRSsOD, patients with CRSwOD exhibited abnormal FC between the OFC and the right middle frontal gyrus.

Abnormal FC was observed between the OFC and the middle frontal gyrus in the CRSwOD group. This may be due to the close relationship between the OFC and the orbitofrontal system and olfaction (23). The middle frontal gyrus is an important region of the orbitofrontal system, which is involved in emotion, social behavior, and autonomic control. Previous studies have suggested that the middle frontal gyrus is involved in attention, working memory, and language processing (24). Activation of the bilateral middle frontal gyrus has been observed in healthy individuals when identifying odor-containing pictures and when presented with olfactory word cues (25). Increased neural activity in the right middle frontal gyrus has been observed during successful odor recognition (26, 27). Positron emission tomography studies have also shown increased regional cerebral blood flow in the middle frontal gyrus and superior frontal gyrus when individuals evaluated the pleasantness and familiarity of odors (28).

In CRSwOD patients, there was a positive correlation between the nasal endoscopy score (LK score), sinus CT score (LM score), and FC values between the right middle frontal gyrus and the left OFC. Higher scores in these evaluations indicate a greater severity of nasal mucosal inflammation and polyp grading, which leads to more severe OD. We speculate that when OD worsen, the FC between the right middle frontal gyrus and the left OFC strengthens to compensate for the olfactory loss caused by the dysfunction. On the other hand, there was a negative correlation between QOD-NS scores and the FC values between the right middle frontal gyrus and the left OFC. Higher QOD-NS scores indicate milder OD, and in these patients, there was no enhanced FC between the right middle frontal gyrus and the left OFC. This may be related to the reciprocal interaction between the middle frontal gyrus and the olfactory network, as the neurons in the middle frontal gyrus are involved in the recognition and processing of olfactory information. As OD worsens, the FC between the middle frontal gyrus and the OFC in the olfactory network may strengthen, and it may weaken when OD improves. Studies have shown that the middle frontal gyrus can modulate olfactory perception and memory based on emotional and mnemonic information (29). These regions are closely connected to the olfactory system and are involved in the regulation of emotion and behavior (30). For example, pleasant odors can evoke positive emotions, while unpleasant odors can evoke negative emotions (31). These emotional reactions and behavioral regulation are closely related to the orbitofrontal system. Additionally, it has been shown that olfactory information is encoded in the olfactory bulbs and transmitted to the piriform cortex. The piriform cortex projects to several brain regions within the limbic system, such as the anterior cingulate cortex and the amygdala, and is directly connected to the frontal cortex through the axonal pathways of the OFC (32). The piriform cortex is known to play a crucial role in odor discrimination (33), and a decrease in FC between the piriform cortex and the middle frontal gyrus has been observed in individuals with decreased olfactory discernment.

## 5 Conclusion

In conclusion, patients with CRSwOD exhibit changes in the brain’s olfactory network, particularly abnormal FC between the core olfactory network (OFC) and the middle frontal gyrus. This abnormal FC may be an important contributor to the OD observed in CRSwOD patients. There is a close relationship between nasal inflammation, polyp severity, and OD and the FC between the middle frontal gyrus and the OFC. Abnormal FC may be a neurobiological mechanism underlying OD in patients with CRSwOD.

There are several limitations in our study. First, it was a cross-sectional study with only one scan per participant. Further longitudinal studies are needed to confirm and further explore our findings to increase our understanding of CRSwOD. Second, the small sample size restricted our ability to investigate changes in other indices. Third, we focused on internetwork relationships and did not explore intranetwork changes. Finally, we did not focus on the current research on olfactory transmission pathways in CRS. Future studies with higher field strength instruments or better preprocessing methods to obtain clearer cortical and subcortical nuclei could provide valuable insights into the mechanisms underlying the transition from CRSsOD to CRSwOD.

## Data availability statement

The original contributions presented in the study are included in the article/supplementary material, further inquiries can be directed to the corresponding author.

## Ethics statement

The studies involving humans were approved by the Ethics Committee of the First Affiliated Hospital of Nanchang University (ethics approval code: (2022) CDYFYYLK (08-017)). The studies were conducted in accordance with the local legislation and institutional requirements. The participants provided their written informed consent to participate in this study.

## Author contributions

YM: Writing – original draft. JJ: Methodology, Writing – original draft. YW: Data curation, Writing – original draft. JX: Formal analysis, Writing – original draft. HL: Software, Writing – original draft. JL: Writing – original draft, Project administration. HK: Writing – original draft, Visualization. XJ: Project administration, Writing – original draft. YC: Conceptualization, Writing – review & editing.
